# Adverse Effects of Cannabinoids and Tobacco Consumption on the Cardiovascular System: A Systematic Review

**DOI:** 10.7759/cureus.29208

**Published:** 2022-09-15

**Authors:** Anas A Abu Jad, Anvesh Ravanavena, Chetna Ravindra, Emmanuelar O Igweonu-Nwakile, Safina Ali, Salomi Paul, Shreyas Yakkali, Sneha Teresa Selvin, Sonu Thomas, Viktoriya Bikeyeva, Ahmed Abdullah, Aleksandra Radivojevic, Prachi Balani

**Affiliations:** 1 Internal Medicine, California Institute of Behavioral Neurosciences & Psychology, Fairfield, USA; 2 General Surgery, California Institute of Behavioral Neurosciences & Psychology, Fairfield, USA

**Keywords:** arrhythmia, tetrahydrocannabinol (thc), sudden cardiac death, cannabinoid, marijuana, cannabis, smoking, tobacco, myocardial infarction, cardio vascular disease

## Abstract

With the recent legalization of marijuana in several countries for recreational use, a controversial belief is spreading about it being "safe". In this systematic review, we decided to investigate this belief and present the adverse effects of marijuana and tobacco smoking on the cardiovascular system.

We carried out an electronic search on databases including PubMed, PubMed Central, and Medline. Medical Subject Headings (MeSH) terms and different keywords were used for data collection. We included studies published in the last 10 years that were in English. All types of study subjects were accepted. Grey literature, books, case reports and case series, overlapping and duplicate studies, and studies older than 10 years were excluded.

In this review, we included 18 studies, which we then separated into the "tobacco and cardiovascular disease" arm and the "cannabinoids and cardiovascular disease" arm. We had 11 and seven studies for each of the arms, respectively. The types of articles included in this review were traditional and systematic reviews and meta-analyses.

After reviewing all the data included in this article, we found out that cannabinoid consumption has a more devastating effect on the cardiovascular system when compared to tobacco. The shocking fact was that in several cases, deadly adverse effects were observed in patients within a few hours after consumption or even during their first time using cannabinoids.

## Introduction and background

Cardiovascular disease (CVD) is a disorder that causes dysfunction and damage to the heart and blood vessels and causes complications [1]. It’s a well-known fact that CVD is the most common cause of death worldwide [1]. Specifically, it causes 25-50% of deaths in developing and even developed countries [1], not to mention the disabilities it causes in the population and the enormous expenses for its treatments [2]. Smoking is the most significant cause of preventable disease and death [2]. It is responsible for seven million deaths per year worldwide [2]. Every year alone, nearly 500,000 people die prematurely in the United States alone [2]. Another 16 million Americans have at least one severe illness caused by smoking [2].

Besides traditional risk factors, tobacco smoking has been considered a significant cause of CVD [1,3,4,5]. Whether it is passive or active smoking, cigarettes, or other nicotine-delivery systems like waterpipes or non-smoke tobacco, they all share most of the harmful effects on the heart and blood vessels [3,4,6]. The mechanism by which tobacco consumption causes this massive damage is multifactorial, ranging from containing many chemicals that disrupt cellular function to the oxidative stress and inflammation of approximately every tissue type in the human body [3,4]. A fact worth adding is that there is no safe amount of cigarette consumption [7]. However, decreasing tobacco consumption will reduce the significance of disease risk and progression to some degree [7].

Marijuana is the most abused illicit drug in the United States and worldwide [8,9]. The recreational use of cannabis and its derivatives is another major global concern, not only because of the harm it causes to one's health but also because of the chemical modifications made to marijuana and the subsequent promotion of these new variants as safer alternatives to the native plant, thereby increasing the number of consumers. Also, the recent legalization of the movement of marijuana and other cannabinoids in different countries for recreational uses and the beneficial pharmacologic effects of some of their types has increased the number of cannabinoid smokers [9]. The factors mentioned above have also created a belief that they are equal to or even less harmful than tobacco.

This article will compare the effects of tobacco smoking versus cannabinoid consumption on the cardiovascular system and the severity of dysfunction they cause. It will also explain the mechanism of damage for each of those risk factors.

## Review

Methods

**Figure 1:**
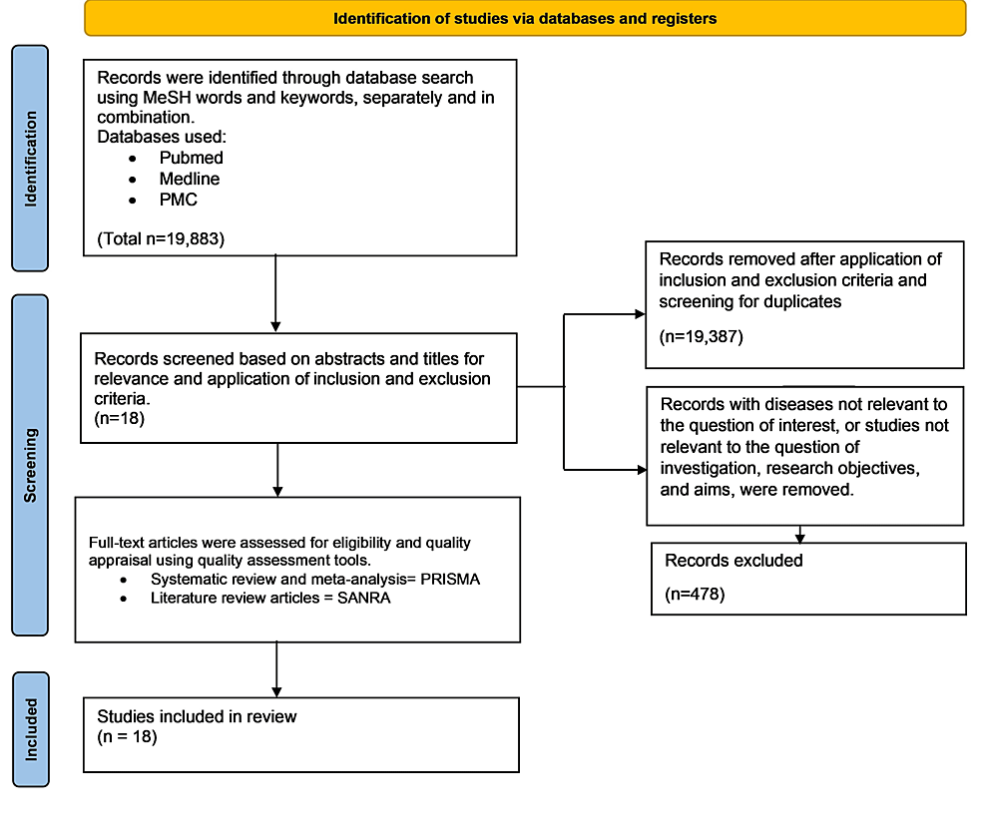
PRISMA flow diagram demonstrating the process of data collection MeSH: Medical Subject Heading; PMC: PubMed Central; PRISMA: Preferred Reporting Items for Systematic Reviews and Meta-Analyses; SANRA: Scale for the Assessment of Narrative Review Articles [10]. This figure is created by the authors.

We followed the Preferred Reporting Items for Systematic Reviews and Meta-Analysis (PRISMA) guidelines for conducting our systematic review [10]. A systematic search was conducted in multiple electronic databases, including PubMed, PubMed Central, and Medline for data collection. We explored the databases by using terms of Medical Subject Heading (MeSH) and keywords like "tobacco," "smoking," "cardiovascular disease," "cannabis," "marijuana," and "myocardial infarction" separately and in combination to find relevant articles. A total of 19,883 records were found in electronic databases, as shown in Figure 1 below.

Inclusion Criteria

We included records in English only. We identified and included studies published in the last 10 years. We had no restrictions on the types of study subjects. Meta-analyses, traditional reviews, and systematic reviews were the records included.

Exclusion Criteria

Grey literature, books, case series, case reports, overlapping studies, duplicate studies, studies in languages other than English, and studies before 2012 were excluded.

Results

In our systematic review, we identified a total of 19,883 records from searches in the databases. No additional records were identified from other sources. After the application of inclusion and exclusion criteria, 19,389 records were removed. After the removal of two duplicates, further screening of abstracts, titles, and whole articles was done, and a total of 18 records were retained. For quality assessment, we set a cutoff point of 70%, and we screened the remaining 18 studies by the following means: systematic review and meta-analysis using PRISMA; literature review articles using Scale for the Assessment of Narrative Review Articles (SANRA).

A total of 18 studies were finally selected to be included in the review. Among the selected studies, 11 of them discussed the relationship between tobacco smoking and CVD with approximately 8,000,000 patients. On the other hand, seven studies were about cannabis and CVD. Overall, we found that cannabis has a different and more devastating way of damaging the cardiovascular system (CVS) when compared to tobacco consumption.

Discussion

Pathophysiology of CVD

**Figure 2:**
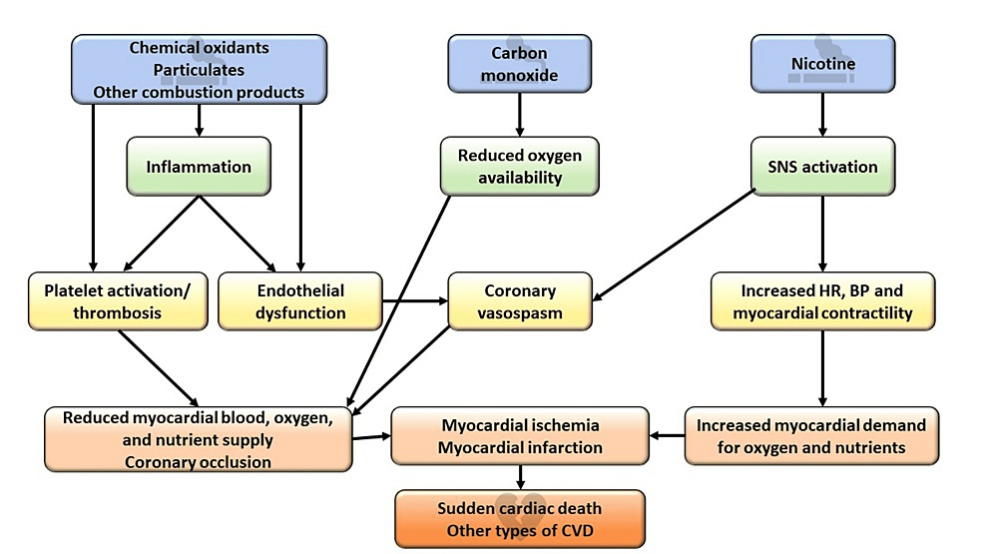
The pathophysiology of CVD CVD: cardiovascular disease; SNS: sympathetic nervous system; HR: heart rate; BP: blood pressure. The figure is created by the authors.

CVD results from several pathological processes, including inflammation, endothelial dysfunction, prothrombotic effects, altered lipid metabolism, and increased demand with decreased oxygen supply (as shown in Figure 2).

Different risk factors play a role in one or more of the mentioned processes. This defines how serious the risk factor is and directs management to decrease the progression and further complications. Tobacco smoking and cannabis use are among the significant modifiable causes of CVD [2].

CVS Damage Due to Tobacco Consumption

Over the last several decades, many studies worldwide have provided evidence of a causative relationship between tobacco consumption and CVD. Since CVD is the leading cause of death globally, complications from tobacco smoking are a priority in government and research organizations’ efforts to help decrease its consequences. Tobacco consumption was shown to cause devastating effects on the cardiovascular system, leading to death and life-long disabilities. Smoking tobacco, which is the most common method of tobacco consumption, endangers not only the smoker but also the people around them, including children. This is known as passive smoking or second-hand smoking.

Most CVDs start with atherosclerosis. Atherosclerosis is the presence of a plaque in a blood vessel’s wall, which causes narrowing and irregularity of the inner layer of the vessel. It reduces artery elasticity and blood flow, reducing blood supply to distal tissues. Ischemia is the disproportion between oxygen demand and supply, which occurs due to decreased blood flow to a specific tissue. Eventually, a plaque can rupture and cause thrombosis, which is the complete occlusion of a blood vessel by a blood clot. Infarction (tissue death) occurs after the deprivation of oxygen and nutrients. In a nutshell, these are the complications in most cases of CVD.

In 2020, Centner et al. found that in experimental mice with a high-fat diet and exposure to tobacco smoking five days a week, the arterial plaque contained components that made it more prone to rupture than those mice not exposed to tobacco [11]. A meta-analysis published in 2018 by Hackshaw et al. aimed to find a dose-dependent relationship between tobacco consumption and CVD. The findings were shocking. Even smoking one cigarette per day causes roughly half the damage seen in patients who smoke 20 cigarettes per day [7]. This means there is no safe amount of smoking.

As some might think, the culprit in smoking-induced damage isn’t just the nicotine but also the chemical ingredients and combustion products. Centner et al., in their study in 2020, also found that exposure of mice to the gas phase of smoke (lacking tar and nicotine) was sufficient to cause oxidative stress and increase total cholesterol [11]. Waterpipe smoking is another method of tobacco consumption that has a reputation for being less harmful than cigarette smoking, given that smoke goes through water before being inhaled and therefore washes out the toxins made by combustion. In this regard, Al Ali et al., in their meta-analysis in 2020, found similar adverse effects of smoking cigarettes or waterpipes [3].

While nicotine isn’t the most harmful component of cigarette smoking, it has mild adverse effects on CVS. Cooke et al., in their study in 2015, have shown that despite nicotine replacement therapy being safe in the short term, it is not entirely known how it affects CVS during long-term use or high-dose use [12], because studies have found adverse effects caused solely by nicotine. Such effects may include transient elevations in blood pressure (being a sympathomimetic agent), endothelial dysfunction, insulin resistance, and dyslipidemia [2].

Other types of smoking-related CVDs that were reviewed include sudden cardiac death, which smokers have a three-fold risk of developing due to smoking, as found in a meta-analysis by Aune et al. in 2018 [13]; atrial fibrillation (AF), which current smokers have an increased risk of developing by more than 30% compared to never smokers [14]; heart failure; and structural remodeling of the heart. A study by Kamimura et al. in 2018 on African American participants has found that smoking leads to a higher risk of structural changes of the left ventricle and a higher risk of hospitalization due to incident heart failure in a dose-dependent manner [4]. Cheng et al., in their meta-analysis in 2013, have found a slightly increased risk of developing venous thromboembolism in smokers [5].

Another problem is that passive smoking (also known as second-hand smoking or environmental tobacco exposure) has the same effect as active smoking on adult participants, as shown by Khoramdad et al. in their meta-analysis in 2019 [1]. While active smoking has debilitating adverse effects on the smoker, passive smokers, especially children, involuntarily suffer similar harm. Concerns have emerged in recent decades following the implementation of highly effective legislation aimed at reducing smoking prevalence, such as the prohibition of smoking in public places. However, family members of smokers are still disadvantaged. Several studies were conducted to investigate the effects of passive smoking on children, and some have found detrimental effects on CVS, causing a risk for stroke [15] and increased systolic blood pressure [6]. Table 1 shows the studies included in this group and their characteristics.

**Table 1:** Included studies and their characteristics in the “Tobacco and CVD” arm CVD: cardiovascular disease; WPS: waterpipe smoking; PS: passive smoking; LV: Left Ventricular; HF: Heart Failure; VTE: venous thromboembolism; BMI: body mass index.

Author	Year	Type of study	Patients	Purpose of study	Result	Conclusion
Centner A. [11]	2020	Review	N/R	The role of tobacco and nicotine in senescence and atherosclerosis.	Nicotine is involved in the process of senescence in cells and the progression of atherosclerosis.	Nicotine increases oxidative stress and the inflammatory burden on vascular cells, which increases the risk of atherosclerosis progression and CVD.
Al Ali R. [3]	2020	Meta-analysis	38,037	The cardiovascular effects of WPS.	WPS has similar effects to those observed in cigarette smoking.	Further studies should be done to scrutinize the long-term effects and benefits of WPS cessation. Waterpipe smokers have the belief that it's not as harmful as cigarette smoking, and this misbelief should be emphasized in investigations.
Khoramdad M. [1]	2019	Meta-analysis	2,324,607	Evaluate the association between PS and CVD.	PS and CVD have a significant association.	Since the association is established, there should be measures and legislation in place to decrease the risk of disease occurrence.
Aryanpur M. [6]	2019	Meta-analysis	192,067	The effect of passive smoking on blood pressure in children and adolescents.	No statistically significant association was found between passive smoking and hypertension in children.	Passive smoking and hypertension in children were found not to be linked, which may be explained by the absence of a dose-dependent effect of smoking.
Aune D. [14]	2018	Meta-analysis	1,316,872	Clarify the association between tobacco smoking and atrial fibrillation.	An association was found between smokers and atrial fibrillation.	There is an increased association between current smokers and atrial fibrillation in a dose-dependent manner. But the association is weaker in former smokers than it is in current smokers.
Aune D. [13]	2018	Meta-analysis	138,273	Clarify the association between tobacco smoking and sudden cardiac death.	An association was found between smoking and sudden cardiac death.	Current smokers have a threefold increase in relative risk of sudden cardiac death, whereas former smokers have a 38% increase in risk.
Hackshaw A. [7]	2018	Meta-analysis	N/R	Quantification of the added risk for each smoked cigarette in light smokers (1-5 cigarettes per day) for coronary heart disease and stroke.	Light smoking is shown to increase the risk of CVD. Reducing the number of cigarettes also helps to lower the risk of cancer and CVD.	There is no safe amount of smoking. Light smoking has a greater risk than is recognized by healthcare professionals or smokers.
Kamimura D. [4]	2018	Review	4,129	Evaluate the association of cigarette smoking with LV dysfunction and HF.	The association between smoking and HF was statistically significant for African American participants.	Cigarette smoking is a major risk factor for LV hypertrophy, systolic dysfunction, and incident HF hospitalization.
Lee PN. [15]	2017	Meta-analysis	N/R	SHS and the risk of stroke in non-smokers.	A statistically significant association was found between SHS and stroke in non-smokers, which increases with increased exposure to SHS.	Non-smokers who are exposed to SHS are at risk of developing a stroke. Further investigations should be done to conclusively establish this relationship.
Cooke JP. [12]	2015	Review	N/R	The adverse effects of nicotine on CVS.	Nicotine has been linked to an increase in the risk and progression of atherosclerosis.	Specific genes and the willingness to be harmed by nicotine have a predictable relationship.
Cheng Y. [5]	2013	Meta-analysis	3,966,184	Evaluate the association between tobacco smoking and VTE.	When compared to non-smokers, smokers were found to have a higher risk of developing VTE.	Although BMI can be a confounding factor, smokers have a higher risk of VTE.

CVS Damage Due to Cannabis Consumption

Cannabis, also known as marijuana, is a plant that has been used for thousands of years for its analgesic and anti-inflammatory effects. The main active components in cannabis are delta-9-tetrahydrocannabinol (THC) and cannabidiol (CBD). The effects these components produce are mediated by the cannabinoid receptors known as CB1 and CB2, which are widespread in mammalian bodies. Endogenous cannabinoids stimulate these receptors, and they play roles in different physiological processes, including neurological and immunological. CB1 is primarily found in the central nervous system, which is believed to be responsible for the psychotropic effect of THC. On the other hand, CB2 is predominantly found in the immune system. Thus, it plays an anti-inflammatory role in the immune modulation process.

Reviewed studies have shown strong evidence of the association between cannabis and different cardiovascular diseases, including:

Myocardial infarction (MI): In 2012, a review by Kimesh et al. found a substantial relationship between marijuana use and acute MI. In investigated acute MI cases, young, otherwise healthy males with a mean age of 31 were found not to have any other risk factors for CVD (hyperlipidemia, diabetes mellitus, family history, previous coronary artery disease, or hypertension) [8,16]. In 80% of cases, patients presented with symptoms of MI within six hours after consumption [8]. Cannabis use was also linked to higher short-term mortality among patients with cannabis-induced MI [17]. This may be due to the analgesic, psychoactive or cardiodepressant effects of marijuana, and thus delayed proper diagnosis and management of MI (thrombolysis and stenting) [17].

Atherosclerosis and ischemic heart disease: Multiple factors play proatherogenic or antiatherogenic roles in atherogenesis. Cannabis consumption stimulates CB1 and CB2, which are found in the heart and blood vessels. These receptors have opposite effects regarding atherogenesis, as CB1 stimulation leads to a proatherogenic effect while CB2 has an antiatherogenic effect [9].

The stimulation of CB1 after cannabis consumption increases the production of reactive oxygen species (ROS), mitogen-activated protein kinases (MAPK), oxidized low-density lipoprotein (LDL), and endothelial damage. This way, CB1 stimulation is believed to participate in the progression of atherosclerosis and further worsen the ischemia of the myocardium [9].

CB2 agonism, on the other hand, has been shown to reduce the inflammatory response to molecules such as endotoxin and tumor necrosis factor (TNF-α). When TNF-α is suppressed, endothelial cells express less intercellular adhesion molecule-1, vascular cell adhesion molecule-1, and monocyte chemoattractant protein-1. Thus, decreased monocyte adhesion to endothelial cells and transendothelial migration of monocytes

Despite the antiatherogenic effect of cannabinoid consumption, studies have shown an overall effect that leads to an increased number of acute coronary syndrome cases, the pathophysiology of which is not known yet [9].

Cerebrovascular disease: Neurological symptoms were the most common reason for hospitalization after cannabis use, while ischemic stroke was the most common among neurological adverse effects [18]. In an Australian general population study, patients who used cannabis during the year before the investigation had a 2.3-fold higher risk of stroke. Participants who used marijuana once a week or more increased their risk by 4.7 times. The reason behind cerebrovascular manifestations is believed to be reversible cerebrovascular spasm, vasculitis, and hypotension with impaired regulation of cerebral blood flow [19].

Cannabis arteritis: It is clinically similar to thromboangiitis obliterans, which is substantially associated with tobacco smoking, but is found to develop earlier with concurrent tobacco smoking and cannabis use. The pathological pattern is the presence of segmental narrowing without a collateral blood supply, which causes progressive ischemia of upper and lower extremity tissue with further development of necrosis and gangrene. Also, this complication was found to be dose-dependent [18].

Synthetic cannabinoids: The chemical modification of cannabis has introduced new compounds with specific properties and adverse effects that are harder to predict. New cannabinoids have shown different contents of the active substance by changing the potency and concentration of specific compounds in consumed cannabinoids. Therefore, it has become problematic to detect cannabinoid levels in regular toxicology screens, making it more challenging to establish the association with adverse effects. Being "legal" alternatives to marijuana has given these compounds popularity, accompanied by an increase in reported adverse effects [19,20].

Table 2 shows the studies included in this group and their characteristics.

**Table 2:** Included studies and their characteristics in the “Cannabis and CVD” arm CVD: cardiovascular disease; MI: myocardial infarction.

Author	Year	Type of study	patients	Purpose of the study	Result	Conclusion
Latif Z. [16]	2020	review	N/R	The physiological effects of marijuana on CVS.	Marijuana is related to several CVDs.	The harm from marijuana consumption is a well-known fact, but several characteristics of a used drug such as potency, way of consumption, and concurrent use of other drugs need to be identified in order to fully explain the relationship.
Puhl S. [17]	2019	Review	N/R	The function of cannabinoid receptors CB1 and CB2 in cardiac physiology and potential therapeutic manipulation in ischemic heart disease.	Interventions targeting the endocannabinoid system have been shown to have a significant impact on the severity, progression, and functional outcome of ischemic heart disease.	CB2 agonism is shown to decrease inflammation, apoptosis, and fibrosis at the site of MI. CB1 antagonism has also been shown to have a beneficial effect on damaged cardiac tissue post-MI.
Singh A. [18]	2018	Review	N/R	Potential cardiovascular effects of cannabinoid consumption.	Reviewed records have found a strong link between cannabinoid consumption and CVD in previously healthy young people.	Although the pathophysiology of cannabinoid consumption isn’t completely established, primary results show a significant role in CVD.
Goyal H. [19]	2017	Review	N/R	The relationship between ECS and the occurrence of CVD.	Cannabis use is related to many acute and chronic CVDs.	Although more research is needed to address the negative effects of cannabis in both recreational and medicinal use, the reviewed studies show a strong link between CVD and cannabis use. There is a potential benefit to using cannabis that needs to be further studied.
Castellanos D. [20]	2016	review	N/R	Familiarize pediatricians and physicians with the effects of cannabis use in young patients.	Synthetic cannabinoids have more dangerous effects than marijuana.	Although it’s hard to suspect the use of synthetic cannabinoids and detect them on regular toxicology screens, physicians must try to be familiar with the expected findings in patients’ presentations.
Chetty K. [8]	2012	Review	N/R	The relationship between cannabis and MI.	Many of the cases studied of MI patients who consumed cannabis were young and previously healthy.	There is a relationship between cannabis use and MI in young, previously healthy patients, especially after a short period of consumption.
Singla S. [9]	2011	Review	N/R	The role of marijuana smoking and related receptors in the development of atherosclerosis and acute coronary syndromes.	There are plenty of studies with animals and human subjects that have contradictory results, which may be caused by the way cannabis is consumed and the involved receptors.	Cannabinoid receptor modulation may have a good impact on the progression of atherosclerosis and acute coronary syndromes, but studies are limited due to clinical and legal reasons.

Limitations

The fact that cannabinoids' use is illegal in some countries makes the estimated numbers of users and the prevalence of its complications far from accurate. Also, the different modified cannabinoids spread among users make it harder for researchers to keep up with investigating the effects these products may cause. As a result, we notice a scarcity of reliable studies on each of the cannabinoids in use. Since they differ significantly in their potency and content, it is necessary to investigate each of them and their impact on the population’s health.

## Conclusions

The objective of our study was to put the belief in marijuana's safety compared to tobacco under the microscope. Therefore, we investigated the relationship between tobacco and CVD (the most common cause of death worldwide) versus marijuana and CVD. And, after reviewing the included records, we discovered that, while marijuana and tobacco both cause CVD, marijuana causes the development of lethal diseases at a faster rate than tobacco, which causes lethal diseases but over a longer period. In some cases, adverse effects were reported after only a few hours of marijuana use, while in others, they were present right away.

The recent movements toward legalizing cannabinoids for recreational use will help increase cannabinoid consumption prevalence and develop a new epidemic that may be worse than tobacco. Synthetic cannabinoids are another major problem since they make it more challenging to predict the damage they cause economically and to populations’ health. Another aspect that limits research in this matter is the low rate of cannabis use disclosure due to fear of legal consequences after reporting their use to healthcare professionals. Therefore, patients should be educated about the confidentiality of patient-doctor reports to help reach the diagnosis and start treatment as soon as possible to decrease complications.

## References

[1] Khoramdad M, Vahedian-Azimi A, Karimi L, Rahimi-Bashar F, Amini H, Sahebkar A (2020). Association between passive smoking and cardiovascular disease: a systematic review and meta-analysis. IUBMB Life.

[2] U.S. Department of Health and Human Services. (2014). The Health Consequences of Smoking: 50 Years of Progress. A Report of the Surgeon General.. https://www.ncbi.nlm.nih.gov/books/NBK179276/pdf/Bookshelf_NBK179276.pdf.

[3] Al Ali R, Vukadinović D, Maziak W (2020). Cardiovascular effects of waterpipe smoking: a systematic review and meta-analysis. Rev Cardiovasc Med.

[4] Kamimura D, Cain LR, Mentz RJ (2018). Cigarette smoking and incident heart failure: insights from the Jackson Heart Study. Circulation.

[5] Cheng YJ, Liu ZH, Yao FJ, Zeng WT, Zheng DD, Dong YG, Wu SH (2013). Current and former smoking and risk for venous thromboembolism: a systematic review and meta-analysis. PLoS Med.

[6] Aryanpur M, Yousefifard M, Oraii A (2019). Effect of passive exposure to cigarette smoke on blood pressure in children and adolescents: a meta-analysis of epidemiologic studies. BMC Pediatr.

[7] Hackshaw A, Morris JK, Boniface S, Tang JL, Milenković D (2018). Low cigarette consumption and risk of coronary heart disease and stroke: meta-analysis of 141 cohort studies in 55 study reports. BMJ.

[8] Chetty K, Lavoie A, Deghani P (2021). A literature review of cannabis and myocardial infarction - what clinicians may not be aware of. CJC Open.

[9] Singla S, Sachdeva R, Mehta JL (2012). Cannabinoids and atherosclerotic coronary heart disease. Clin Cardiol.

[10] Page MJ, McKenzie JE, Bossuyt PM (2021). The PRISMA 2020 statement: an updated guideline for reporting systematic reviews. BMJ.

[11] Centner AM, Bhide PG, Salazar G (2020). Nicotine in senescence and atherosclerosis. Cells.

[12] Cooke JP (2015). New insights into tobacco-induced vascular disease: clinical ramifications. Methodist Debakey Cardiovasc J.

[13] Aune D, Schlesinger S, Norat T, Riboli E (2018). Tobacco smoking and the risk of sudden cardiac death: a systematic review and meta-analysis of prospective studies. Eur J Epidemiol.

[14] Aune D, Schlesinger S, Norat T, Riboli E (2018). Tobacco smoking and the risk of atrial fibrillation: a systematic review and meta-analysis of prospective studies. Eur J Prev Cardiol.

[15] Lee PN, Thornton AJ, Forey BA, Hamling JS (2017). Environmental tobacco smoke exposure and risk of stroke in never smokers: an updated review with meta-analysis. J Stroke Cerebrovasc Dis.

[16] Latif Z, Garg N (2020). The impact of marijuana on the cardiovascular system: a review of the most common cardiovascular events associated with marijuana use. J Clin Med.

[17] Puhl SL (2020). Cannabinoid-sensitive receptors in cardiac physiology and ischaemia. Biochim Biophys Acta Mol Cell Res.

[18] Singh A, Saluja S, Kumar A, Agrawal S, Thind M, Nanda S, Shirani J (2018). Cardiovascular complications of marijuana and related substances: a review. Cardiol Ther.

[19] Goyal H, Awad HH, Ghali JK (2017). Role of cannabis in cardiovascular disorders. J Thorac Dis.

[20] Castellanos D, Gralnik LM (2016). Synthetic cannabinoids 2015: an update for pediatricians in clinical practice. World J Clin Pediatr.

